# Infertilidade Feminina: Fator de Risco para Doenças Cardiovasculares ou Marcador de uma População com Risco Cardiovascular?

**DOI:** 10.36660/abc.20250250

**Published:** 2026-06-12

**Authors:** Maria Alayde Mendonça Rivera, Ivan Romero Rivera, Glaucia Maria Moraes de Oliveira

**Affiliations:** 1 Universidade Federal de Alagoas Maceió AL Brasil Universidade Federal de Alagoas, Maceió, AL – Brasil; 2 Santa Casa de Misericórdia de Maceió AL Brasil Santa Casa de Misericórdia de Maceió, AL – Brasil; 3 Universidade Federal do Rio de Janeiro Rio de Janeiro RJ Brasil Universidade Federal do Rio de Janeiro, Rio de Janeiro, RJ – Brasil

**Keywords:** Infertilidade, Fator de Risco, Doença Cardiovascular, Sexo Feminino

Segundo a Organização Mundial da Saúde, aproximadamente 17,5% da população adulta (1 em cada 6 pessoas ao redor do mundo) é afetada pela infertilidade, definida como a incapacidade de conceber após 12 meses de relações sexuais regulares sem proteção.^1^ A prevalência varia pouco entre as regiões mundiais, com taxas semelhantes em países de alta, média e baixa renda, constituindo um problema global de saúde pública.^1^

Aproximadamente 85% dos casais inférteis têm uma causa identificável para a infertilidade, enquanto os 15% restantes apresentam infertilidade inexplicada. Conforme ilustrado na Figura 1, a infertilidade pode ser atribuída a múltiplas causas e fatores de risco, cujo tratamento pode eventualmente contribuir para a fertilidade do casal.^1^

**Figura 1:**
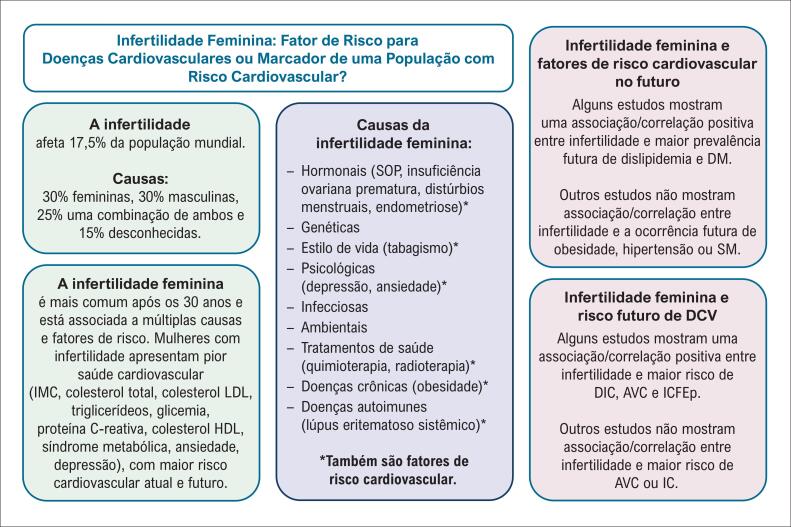
Infertilidade feminina e risco cardiovascular. AVC: acidente vascular cerebral; DIC: doença isquêmica do coração; DM: diabetes mellitus; FOP: falência ovariana prematura; HDL: lipoproteína de alta densidade; IC: insuficiência cardíaca; ICFEp: insuficiência cardíaca com fração de ejeção preservada; IMC: índice de massa corporal; LDL: lipoproteína de baixa densidade; SM: síndrome metabólica; SOP: síndrome dos ovários policísticos.

As doenças cardiovasculares (DCV) são responsáveis, em média, por 30% da mortalidade global em homens e mulheres e abrangem um amplo espectro de doenças, com diferentes formas e graus de apresentação, incluindo doença isquêmica do coração (morte súbita cardíaca, infarto agudo do miocárdio, angina instável, angina estável); doença cerebrovascular (acidente vascular cerebral isquêmico e hemorrágico, ataque isquêmico transitório, hemorragia subaracnoide); doença vascular periférica; insuficiência cardíaca (com fração de ejeção preservada, intermediária e reduzida); e fibrilação atrial.^2^

Esse grupo de doenças compartilha diversos fatores de risco comuns a homens e mulheres (envelhecimento, histórico familiar, hipertensão, diabetes, hipercolesterolemia, tabagismo), além de outros que são exclusivos das mulheres (menarca precoce, distúrbios menstruais, falência ovariana prematura, síndrome dos ovários policísticos, endometriose) ou que têm maior impacto nas mulheres (depressão, ansiedade).^2^

Em mulheres, observa-se que diversos fatores de risco para infertilidade (tabagismo, obesidade, síndrome dos ovários policísticos, falência ovariana prematura, distúrbios menstruais, doenças autoimunes, endometriose) também estão relacionados ao desenvolvimento de DCV, tornando necessário expandir e aprofundar a pesquisa nessa área, considerando a multiplicidade de causas de infertilidade, bem como a multiplicidade de causas e expressão clínica das DCV.^1–4^

Com base em estudos observacionais, a infertilidade tem sido considerada um marcador para o desenvolvimento de doenças imunológicas, câncer e DCV, bem como para uma menor expectativa de vida em homens e mulheres, embora não esteja claro se essa tendência ocorre por razões relacionadas ao estilo de vida (por exemplo, maior consumo de álcool, tabagismo ou hábitos alimentares menos saudáveis), mecanismos biológicos ou uma interação entre ambos.^5^

Em homens, a infertilidade está associada a uma maior incidência de câncer testicular, de próstata, de bexiga e de tireoide; melanoma; linfoma; doença isquêmica do coração; diabetes; e hipertensão.^6^

Em mulheres, a infertilidade aumenta a incidência de câncer de mama, de ovário e de endométrio. Quando associada à síndrome dos ovários policísticos, a infertilidade parece aumentar a incidência de hipertensão (após os 45 anos de idade), hipercolesterolemia e diabetes mellitus (antes dos 45 anos). Outras alterações hormonais relacionadas à infertilidade (irregularidades menstruais, falência ovariana prematura, abortos espontâneos) podem estar associadas à ocorrência futura de DCV, na forma de doença isquêmica do coração, acidente vascular cerebral e insuficiência cardíaca.^7–9^

Não existem ensaios clínicos sobre infertilidade e o risco futuro de DCV. Estudos observacionais transversais mostraram que mulheres diagnosticadas com infertilidade apresentam pior saúde cardiovascular, com maior índice de massa corporal; níveis mais elevados de colesterol total, colesterol LDL, triglicerídeos, glicose sanguínea e proteína C-reativa; e níveis mais baixos de colesterol HDL,^7–9^ bem como maior prevalência de síndrome metabólica^9^ e condições de saúde mental, como ansiedade e depressão.^1^ Tal perfil contribui independentemente para o risco futuro de DCV.^3^

Quanto ao desenvolvimento futuro de DCV em mulheres diagnosticadas com infertilidade, os resultados continuam inconsistentes. Com base em estudos observacionais longitudinais, com acompanhamento variando de 7 a 28 anos, há relatos de que a infertilidade está associada a uma maior prevalência de dislipidemia e diabetes, bem como a um maior risco futuro de doença isquêmica do coração, acidente vascular cerebral e insuficiência cardíaca com fração de ejeção preservada. Outros estudos não mostraram associação entre infertilidade e a ocorrência futura de obesidade, hipertensão, síndrome metabólica, insuficiência cardíaca e acidente vascular cerebral.^7–9^

Considerando a alta prevalência de infertilidade feminina na população mundial,^1^ a frequente associação dessa condição com outros fatores de risco cardiovascular,^2–5^ e a falta de dados sobre o impacto a longo prazo do tratamento hormonal da infertilidade no risco materno futuro de DCV,^10^ são necessários ensaios clínicos delineados para abordar essas questões, a fim de expandir o atendimento cardiovascular às mulheres.

## Data Availability

Os conteúdos subjacentes ao texto da pesquisa estão contidos no manuscrito.
