# Supplementary material for: Infertilidade Feminina: Fator de Risco para Doenças Cardiovasculares ou Marcador de uma População com Risco Cardiovascular?
Source: Arq Bras Cardiol. 2026 Jun 12;123(5):e20250250. [Article in Portuguese] doi: 10.36660/abc.20250250 (PMC13399457; doi:10.36660/abc.20250250)
Supplement: Translation

# Translation: Female Infertility: Cardiovascular Disease Risk Factor or Marker of a Population at Cardiovascular Risk?

**DOI:** 10.36660/abc.20250250i

**Authors:** Maria Alayde Mendonça Rivera, Ivan Romero Rivera, Glaucia Maria Moraes de Oliveira

**Affiliations:** 1 Maceió AL Brazil Universidade Federal de Alagoas, Maceió, AL – Brazil; 2 AL Brazil Santa Casa de Misericórdia de Maceió, AL – Brazil; 3 Rio de Janeiro RJ Brazil Universidade Federal do Rio de Janeiro, Rio de Janeiro, RJ – Brazil

**Keywords:** Infertility, Risk Factor, Cardiovascular Disease, Female Sex

According to the World Health Organization, approximately 17.5% of the adult population (1 in every 6 people in the world) suffers from infertility, defined as the inability to conceive after 12 months of regular unprotected sexual intercourse.^1^ The prevalence varies little between regions, with similar rates in high-, middle-, and low-income countries, constituting a global public health problem.^1^

Approximately 85% of infertile couples have an identifiable cause for infertility, whereas the remaining 15% have unexplained infertility. As shown in the Figure 1, infertility can be attributed to numerous causes and risk factors, the treatment of which can eventually contribute to the couple's fertility.^1^

**Figure 1:** Female infertility and cardiovascular risk. BMI: body mass index; DM: diabetes mellitus; HDL: high-density lipoprotein; HF: heart failure; HFpEF: heart failure with preserved ejection fraction; IHD: ischemic heart disease; LDL: low-density lipoprotein; MS: metabolic syndrome; PCOS: polycystic ovary syndrome; POF: premature ovarian failure.

Cardiovascular disease (CVD) accounts for, on average, 30% of global mortality in men and women and encompasses a broad spectrum of diseases, with different forms and degrees of presentation, including ischemic heart disease (sudden cardiac death, acute myocardial infarction, unstable angina, stable angina); cerebrovascular disease (ischemic and hemorrhagic stroke, transient ischemic attack, subarachnoid hemorrhage); peripheral vascular disease; heart failure (with preserved, intermediate, and reduced ejection fraction); and atrial fibrillation.^2^

This group of diseases shares numerous risk factors common to men and women (aging, family history, hypertension, diabetes, hypercholesterolemia, smoking), in addition to others that are exclusive (early menarche, menstrual disorders, premature ovarian failure, polycystic ovary syndrome, endometriosis) or have a greater impact on women (depression, anxiety).^2^

In women, it is observed that numerous risk factors for infertility (smoking, obesity, polycystic ovary syndrome, premature ovarian failure, menstrual disorders, autoimmune diseases, endometriosis) are also related to the development of CVD, making it necessary to expand and deepen research in this field, considering the multiplicity of causes of infertility, as well as the multiplicity of causes and clinical expression of CVD.^1–4^

Based on observational studies, infertility has been considered a marker for the development of immunological diseases, cancer, and CVD and for a shorter life expectancy in men and women, although it is unclear whether this propensity occurs for reasons related to lifestyle (for example, greater alcohol consumption, smoking, or unhealthy eating habits), biological mechanisms, or an interaction of both.^5^

In men, infertility is associated with a higher incidence of testicular, prostate, bladder, and thyroid cancer; melanoma; lymphoma; ischemic heart disease; diabetes; and hypertension.^6^

In women, infertility increases the incidence of breast, ovarian, and endometrial cancer. When associated with polycystic ovary syndrome, infertility appears to increase the incidence of hypertension (after 45 years of age), hypercholesterolemia, and diabetes mellitus (before 45 years). Other hormonal changes related to infertility (menstrual irregularities, premature ovarian failure, spontaneous abortions) may be associated with the future occurrence of CVD, in the form of ischemic heart disease, stroke, and heart failure.^7–9^

There are no clinical trials on infertility and the future risk of CVD. Cross-sectional observational studies have shown that women diagnosed with infertility have worse cardiovascular health, with higher body mass index; higher levels of total cholesterol, LDL-cholesterol, triglycerides, blood glucose, and C-reactive protein; and lower levels of HDL-cholesterol,^7–9^ as well as a higher prevalence of metabolic syndrome^9^ and mental health conditions such as anxiety and depression.^1^ This profile contributes independently to the future risk of CVD.^3^

Regarding the future development of CVD in women diagnosed with infertility, there are still inconsistent results. Based on longitudinal observational studies, with follow-ups ranging from 7 to 28 years, there are reports that infertility is associated with a higher prevalence of dyslipidemia and diabetes, as well as a higher future risk of ischemic heart disease, stroke, and heart failure with preserved ejection fraction. Other studies have shown no association between infertility and the future occurrence of obesity, hypertension, metabolic syndrome, heart failure, and stroke.^7–9^

Considering the high prevalence of female infertility in the world population,^1^ the frequent association of this condition with other cardiovascular risk factors,^2–5^ and the lack of data on the long-term impact of hormonal infertility treatment on the future maternal risk of CVD,^10^ there is a need for clinical trials designed to address these questions, in order to expand women's cardiovascular care.

## Data Availability

The underlying content of the research text is contained within the manuscript.
